# Replacing the Orchestra? – The Discernibility of Sample Library and Live Orchestra Sounds

**DOI:** 10.1371/journal.pone.0158324

**Published:** 2016-07-06

**Authors:** Reinhard Kopiez, Anna Wolf, Friedrich Platz, Jan Mons

**Affiliations:** 1 University of Music, Drama and Media, Hanover, Germany; 2 State University of Music and Performing Arts, Stuttgart, Germany; Johns Hopkins University, UNITED STATES

## Abstract

Recently, musical sounds from pre-recorded orchestra sample libraries (OSL) have become indispensable in music production for the stage or popular charts. Surprisingly, it is unknown whether human listeners can identify sounds as stemming from real orchestras or OSLs. Thus, an internet-based experiment was conducted to investigate whether a classic orchestral work, produced with sounds from a state-of-the-art OSL, could be reliably discerned from a live orchestra recording of the piece. It could be shown that the entire sample of listeners (*N* = 602) on average identified the correct sound source at 72.5%. This rate slightly exceeded Alan Turing's well-known upper threshold of 70% for a convincing, simulated performance. However, while sound experts tended to correctly identify the sound source, participants with lower listening expertise, who resembled the majority of music consumers, only achieved 68.6%. As non-expert listeners in the experiment were virtually unable to tell the real-life and OSL sounds apart, it is assumed that OSLs will become more common in music production for economic reasons.

## Introduction

The invention of pre-recorded orchestra sample libraries (OSL), as a modern approach to sound synthesis, can be regarded as a minor music-industrial revolution (p. 84 in [1]) which enables the recording of music without the presence of live musicians. Nowadays, pre-recorded and stored musical sounds from OSLs are indispensable to the modern production of various musical genres, for example, in the inexpensive production of demo songs [2] or computer game music [3]. Such sound production frees music producers from the constraints of a professional recording studio or expensive digital equipment [4, 5]. However, outside the world of popular music the question of how sounds stemming from OSLs should be used for the production of classical music is sometimes dominated by ideology. Contrary to critics, even expert listeners had difficulties discerning between a Beethoven Symphony based on sounds from an OSL when compared to a live orchestra recording (LOR) [6]. Other discussions in the field are dominated by such disputes as whether the orchestra part of Wagner’s “The Ring of the Nibelung” may be emulated by a digital orchestra [7], whether this technology will replace orchestra musicians and should be boycotted [8], or whether the virtual orchestra is aesthetically acceptable at all [9]. From the perspective of applied basic research, reliable knowledge about the discernibility between classical music based on OSL versus LOR sounds would be of great importance for the aesthetic acceptance and efficient application of OSL sounds in music production.

But which timbre cues can help listeners discern between OSL and LOR sounds? And, by implication, which sound features are the prerequisites to make an emulated instrument sound natural? Previous research on a direct comparison of natural vs. emulated instrument sounds is rare, but in one of the very few studies [10] a direct comparison of identification rates for 11 instrument sounds based on different sound emulation principles revealed the highest average correct responses for natural sounds (75%), followed by sample-based emulations (58%), hybrid emulations (sample-based plus additive synthesis; 43%), and FM synthesis-based sounds (44.5%). Although sample-based emulations came very close to the natural sounds in terms of evaluation, missing time-variant characteristics in the steady-state phase of an instrument sound remained a shortcoming of emulation principles. Following up on this, studies on the evaluation of wind instrument sound emulations [11, 12] could show that emulated bassoon and oboe sounds with additional source-oriented vibrato modulations were evaluated nearly as clearly as real instrument sounds. Finally, when listeners compared actual, human performances with rendered performances of Baroque music, timing and dynamics were not the most important factors in determining the quality of rendered versions. Instead, evaluations were more influenced by the sound quality of the samples used for the rendered versions [13].

The assumption that listening expertise has an influence of on the discrimination performance between OSL and LOR version is in line with previous research on similar effects of increased domain-specific discrimination performance of the auditory system in highly trained groups. For example, although musical experts and non-experts show similar processing routines for music, highly trained musicians are characterized by a higher signal-to-noise ratio in their answering behavior [14]. In a study based on event-related brain potentials in the spatial resolution of sounds [15, 16], experienced professional conductors showed enhanced auditory localization mechanisms in peripheral space when compared to non-musicians and pianists; repeated listening to very short speech sounds (10 ms of vowels) [17] showed that the scores of phonetically untrained participants (students) ranged from 15 to 25% below those of the phonetically trained (graduate students and faculty with phonetical training between 1 and 5 yrs.). However, rapid learning took place in both groups and reached saturation in the phonetically trained group for selected vowels after 4–5 blocks of 36 vocal stimuli each. In a study on the effects of extensive training (more than 20,000 repeated sequences) on the identification of the temporal order of three contiguous frequencies [18], participants achieved above-chance identification with a duration threshold of only 2–7 ms for single tones. The effect of extensive training (6–10 hrs with 7,000–9,000 trials) on sound discrimination learning (e.g., frequency or duration discrimination) increased performance in various auditory discrimination tasks significantly [19]. To summarize, auditory perceptual learning by long-term involvement of sounds can significantly increase the discrimination of sound features.

To this end, in a psychophysical discrimination task we measured the sensitivity to sounds from OSL and LOR versions of orchestra music and the influence of listening expertise on discrimination performance.

## Materials and Methods

### General Methodological Considerations

The more general theoretical framework was derived from Turing's criterion for artificial intelligence (AI): In his theoretical treatise, *The Imitation Game* [20], the questions of an interrogator are answered by either a human or a machine. AI was said to be present when the interrogator could correctly identify the source of the response only 70% of the time or less. In the current experiment, the efficiency of an OSL was confirmed if the listener’s correct response rate (average of hits and correct rejections) was less than 70%.

As a psychological measurement paradigm, the Signal Detection Theory (SDT) was used. Its basic idea is that a description of participants’ response behavior in a psychophysical discrimination task as a function of correct answers only (so-called “hits”) must remain incomplete. Instead, people’s accuracy in discrimination tasks should be related to four decision alternatives: as the result of correct responses (hits and correct rejections) and errors (misses and false alarms) [21]. Following from this, people’s discrimination performance is high when they have a much higher correct response rate compared to the error rate. The corresponding indicator of a person’s sensitivity and performance in a discrimination task is the so-called “*d* prime” (*d*’) value, which is calculated by Eq 1 on the basis of *z* transformed relative answer proportions (see p. 8 in [21]):

d′=z(Hit rate)−z(False alarm rate) (1)

The second indicator of a person’s discrimination performance is the response bias *c*, which describes a participant’s tendency to respond on some basis other than merit, showing a tilt toward one response or the other (see p. 27 in [21]). This bias is measured by the index *c* according to Eq 2:

c=−0.5⋅(z(Hit rate)+z(False alarm rate)) (2)

Finally, reflections on a priori test power resulted in the following calculation of the sample size: Assuming a small effect size of *d*’ = 0.5 (for benchmarks, see [22]), a power of 1-*β* = 0.80 and a significance level of *α* = .05, a sample size of at least *N* = 94 would be required (see p. 25 in [23]) for the discrimination between OSL and LOR sounds in a 2-alternative forced-choice setting. A desirable stronger power of 1-*β* = 0.90 would require a sample size of at least *N* = 122 (see p. 380 in [22]). Since it was unlikely that this high number of participants could be reached by a laboratory study, the data collection was conceived as an internet experiment.

### Stimuli

High-quality imitations of selected phrases of a LOR of Stravinsky's large orchestra work *The Rite of Spring* [24] were produced by means of sounds from the Vienna Symphonic Library (VSL), an orchestra sample library of outstanding quality, to test whether OSL and LOR sound sources are discernible above chance.

Generally, the impressive quality of this extensive library collection with currently more than 2 million sound samples is based on the idea of extensive sound parameter control (for a detailed description see http://www.vsl.co.at). Therefore, various principles have been applied: (a) All sounds have been recorded in an anechoic room with an extremely low-noise floor (the so-called silent stage). (b) Pitches are based on real note-by-note recordings in full range instead of the use of a limited number of recorded pitches which have been transposed. (c) All events are performed with different playing styles or articulations (for example, notes from string instruments are performed in legato, pizzicato, con sordino, or with/without vibrato or note repetition) and are not the result of manipulations by MIDI controller (demonstrations of the different playing styles at the example of violins can be obtained from https://www.youtube.com/watch?v=f-Jnq0nqxzQ). (d) Different sound levels are based on true differences in performed loudness (for example, the timpani samples are based on a maximum of 8 velocity levels covering a dynamic range between *ppp* and *fff*). (e) Transitions between single pitches are controlled by a cross-fade algorithm (performance legato tool) which produces the impression of a natural sounding instrument playing. (f) Groups of instruments are not simply based on multiplied tracks of single-instrument recordings but use real ensemble recordings of groups of varying sizes (for example, the so-called “dimension strings” are comprised of 24 instruments from the string section which have been recorded in homogenous groups with one microphone per instrument and controller access to every single instrument (an illustration of the subtleties of the ensemble recordings for the strings section [so-called dimension strings] can be obtained from https://www.youtube.com/watch?v=BoH2ohPlIGQ). (g) After the sequencer arrangement has been completed, the anechoic sounds are vitalized with a sophisticated convolution reverb (the so-called Multi Impulse Response Stage). (h) Finally, the top-down control of an entire song arrangement is coordinated by the Vienna Ensemble Plugin collection which guarantees the control over every single instrument, the balance between instrument groups, and the intended spatial information. Thus, it is not surprising that reviewers of the library’s first edition confirm the library’s outstanding quality and conclude that the VSL is “one of the most important products of modern music technology” [25] which “makes the samples serve the music” [26].

The choice of Igor Stravinsky's work for large orchestra, *The Rite of Spring*, from 1913 was motivated by three reasons: (a) It contains a great variety of instrument combinations and playing techniques (e.g., pizzicato and flageolet); (b) it is available as a pre-edited MIDI file which is used by the VSL company for the demonstration of the OSL's capacity for high-quality sound [27]; (c) there is an up-to-date, high-quality recording of the piece [24]. The selected sections had a full orchestra sound and featured groups of instruments but no solo instruments. Passages were only selected from the LOR if no interfering noise (e.g., breathing noises from wind instruments) was audible. Finally, 10 sections with a length between 18 and 31 seconds were selected (see Table B in S2 File).

The objective for the audio engineer was the production of the best possible imitation of the LOR version by means of the OSL. In the first step, the score was imported in MIDI format (single tracks for each instrument) to the sequencer program Cubase (V 7.5) for further editing. The control of the VSL plug-in was based on the VSL Vienna Super Package and the Vienna Software Package (sample rate = 44.1 kHz/24 bit). In the second step, selected sections from the LOR source were synchronized with the OSL tracks during the construction of the imitation. Reverb was based on the "MIR Pro Teldex Studio Berlin" environment (Teldex Scoring Stage).

After completion of the first OSL version, the ongoing iterative optimization (Fig B in S1 File) was guided by 3 professional conductors who received sound files of the 10 sections. They listened via high-quality headphones (Sennheiser HD 25 II with audio interface [Native Instruments Komplete Audio 6], no headphone equalization activated) to a comparison between the OSL and LOR and were asked to evaluate the OSL product/performance according to 3 questions:

1. *If you were the conductor of this orchestra*, *how would you comment on the rehearsal*?
2. *Please identify those acoustical features that might identify this work as an OSL product/performance*.
3. *What constitutes the difference between the OSL and the LOR performance in each section*?

Comments and suggestions (e.g., the balance of loudness between instruments, articulation) were protocoled and used by the audio engineer for the next step of optimization. In total, this cycle of iterative optimization was repeated three times until the imitation of the LOR achieved the highest quality. The optimum quality of iterative optimization was reached when no more suggestions were given by the conductors on how to improve the VSL productions/performances from their point of view. An example of the conductors’ comments on the two iterative versions is shown in Table A in S2 File. Sound examples for the illustration of the subtle changes in the process of iterative optimization of passages from Stravinsky’s *The Rite of Spring* based on the OSL sounds can be obtained from https://osf.io/n48x9.

In a third step, the OSL and LOR final versions were matched for average loudness and loudness over the course of time (see Fig C in S1 File). In a fourth step, final versions of the selected sections were provided with fade-in/fade-out, exported in WAV format (44.1 kHz/16 bit) and converted to a very high-quality mp3 format (320 kBit/s) as determined by the technical requirements of the online survey provider. All steps of the OSL audio production were set down in a protocol by the audio engineer.

### Procedure

The study was conceived as an online experiment (platform *SoSci Survey*, see http://www.soscisurvey.de), in which participants were asked to listen to a randomized presentation of 20 short music examples (2 × 10 versions for OSL and LOR, respectively) with durations between 18 and 30 seconds in a single-choice paradigm (choices: computer/orchestra/don't know). Answers were coded according to the scheme shown in Fig D in S1 File.

First, participants were instructed to use headphones and to de-activate the sound effects on their local PC. To control for the loudness level, a short classical piece with the same loudness level as the subsequent stimuli was played, which participants were supposed to adjust to a comfortable loudness level. For a manipulation check [28–30], and to guarantee for a minimum of loudness required for soft passages, participants had to type in how many timpani beats (*n* = 7) they heard in a short experimental stimulus. In the case of a correct answer, participants could proceed to the next page or they had to readjust the loudness level. If they repeatedly identified the number of timpani beats incorrectly, their participation was terminated. Participants received the following instructions: *"You will now listen to some short musical examples*. *Please answer after each example whether you think this performance was produced by a live orchestra or by computer-controlled sounds*. *After the experiment you will see the percentage of correct responses*.*"* Sound examples started automatically. The presentation of the stimuli was completely randomized for each participant, and after each stimulus the answer sheet appeared. Examples could only be listened to once, as the forward/backward functions of the internet browser were deactivated during the complete study. To control for answer reliability, two stimuli (one OSL and one LOR version) were presented for retesting. Additionally, participants could give information on their evaluation strategies in an open answer field at the end of the online study.

Finally, the following control variables were recorded before and after the experiment: (a) familiarity with the composition (at the end, participants were asked to type in the name of the composition and composer when indicating familiarity); (b) musical background as measured by the General Musical Sophistication factor of the Goldsmiths Musical Sophistication Index (*Gold-MSI*) [31] (Sophistication is used in terms of a psychometric construct that refers to musical skills resulting from formal and informal training. Persons with higher levels of musical sophistication were expected "to respond to a greater range of musical situations, are more flexible in their responses, and possess more effective means of achieving their goals when engaging with music" (p. 2 in [31]); (c) self-reported level of sound discrimination expertise was categorized into 5 occupational groups (non-musician/amateur musician, music teacher/musicologist, conductor/orchestra musician, music producer/audio engineer, composer/arranger). The total participation time was about 15 minutes.

### Participants

The study was conducted in July and August 2014. The Ethics Board of the Hanover University of Music, Drama and Media approved the research undertaken and reported in the manuscript. Participants were invited via a web link that was circulated to mailing lists, Facebook groups, forums and blogs of music producers, film music composers, arrangers, orchestra musicians, and musicologists. All individuals in this manuscript have given written informed consent (as outlined in the PLOS consent form) to publish these case details. In total, *N* = 1,563 participants logged onto the website. Valid cases were identified stepwise (see Fig A in S1 File). To control for the reliability of responses [28–30], we removed those cases with incomplete questionnaires, multiple participation (identified by same IP address) or implausible participation time from the data set. In a second step, those respondents with more than 4 responses in the category of "don't know" and more than 1 (out of 2) unreliable answers for the re-test items were deleted. Consequently, participants with no more than 3 "don't know" responses remained in the data sat. However, the participants’ mean Hit/False alarm rate was calculated without those “don’t know” answers. This resulted in a total of *N* = 602 valid cases (mean age was 32.31 yrs, *SD* = 10.93, 70% male; see Table B in S2 File for sample characteristics).

### Data Analysis

For the calculation of the discrimination performance indicator *d prime* (*d*‘), an Excel spreadsheet [32] was used for signal detection analysis (SDT; for the coding of answers and calculation of SDT parameters see Fig D in S1 File). The following coding scheme for the given answers was used: If a VSL performance was correctly identified, the answer was coded as "hit", whereas if a LOR performance was identified as VSL, the answer was coded as "false alarm". The calculation of *d'* and the calculation of the bias *c* followed the common rules (see Eqs 1 and 2). To avoid infinite *d*’ values, answer proportions of 0% and 100% were converted in line with the standard recommendations (p. 8 in [21]: A response rate of 0% was corrected to the constant value of 1/(2 *N*) and a rate of 100% to the value of 1-1/(2 *N*) with *N* = 20 (2 × 10 stimuli). For example, a *Hit rate* of 100% (= 1.0) was corrected to a value of .975 and a *Hit rate* of 0% to a value of .025.

**Fig 1:**
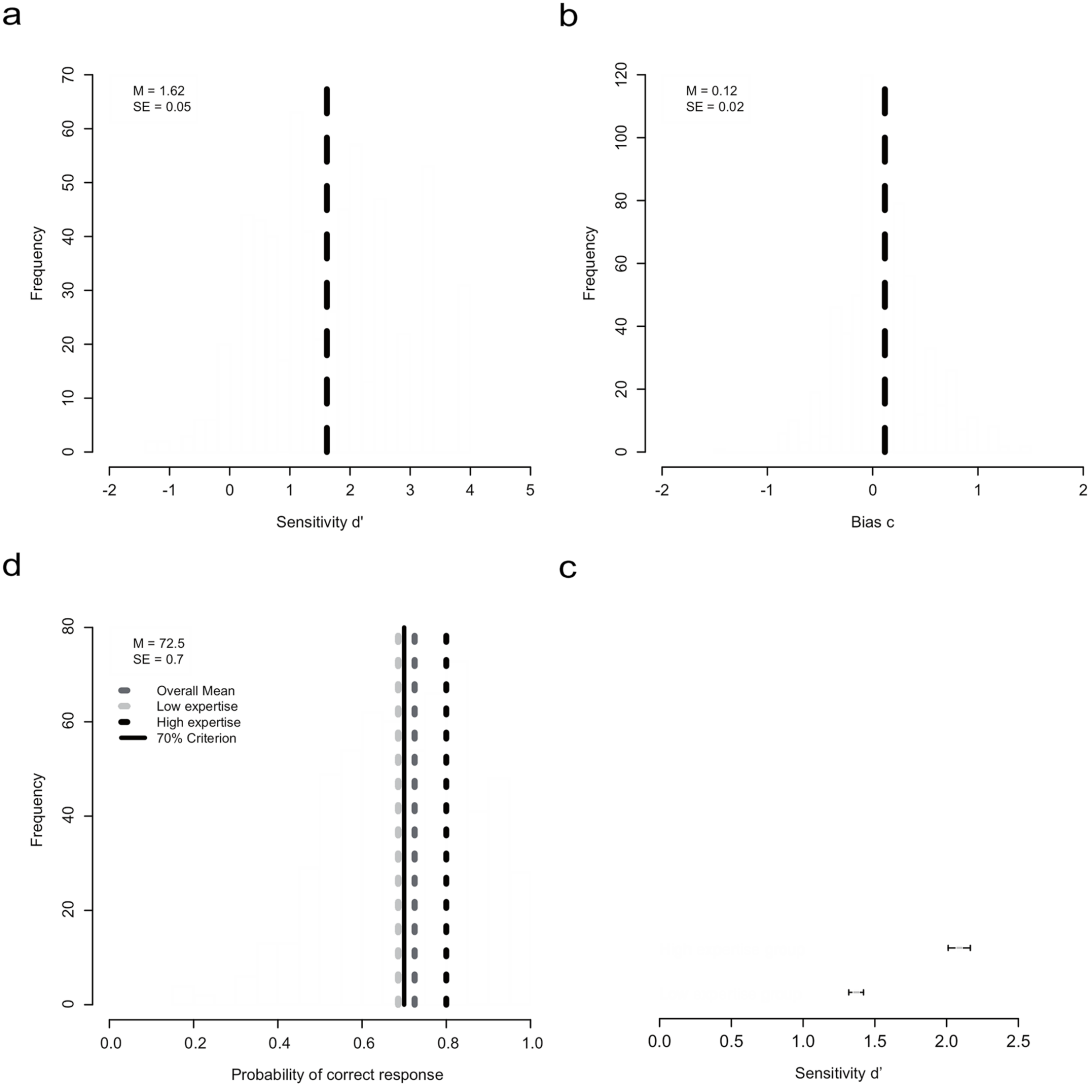
Results from the auditory discrimination task between orchestra sample library and live orchestra recording. (a) Histogram of the overall sensitivity (*N* = 602) in the discrimination of orchestra sample libraries (OSL) and live orchestra recordings (LOR) in a single-choice paradigm. The dashed line represents the mean discrimination performance, whereas the value of 0 indicates discrimination at chance level. (b) Distribution of response bias with a mean close to 0. Negative values indicate answering response in favor of the OSL; the positive values indicate answering response in favor of the LOR. (c) Discrimination performance for groups of low sound-discrimination expertise (non-musicians, amateur musicians, musicologists, and music teachers) and high sound-discrimination expertise (orchestra musicians, audio engineers, conductors, composers, and arrangers). (d) Correct response rates (hits and correct rejections) for the total sample (72.5%), the subgroups of low vs. high sound discrimination expertise (68.6% vs. 80.0%), and Turing's criterion of 70% for correctly identifying the sound sources to prove AI. Only the group with low sound-discrimination expertise was “cheated” more easily by the samples in that they could not identify correctly the sound source above a rate of 70%.

After calculation of the SDT values (Table D in S2 File), group analyses of sound discrimination performance for the 5 a priori groups (differing in sound-discrimination expertise as indicated by self-reported occupation) were conducted by means of an omnibus ANOVA procedure (Table E in S2 File). The ANOVA revealed a significant overall between-groups difference (*F*(4) = 17.35, *p* < .001, *η*^2^ = 0.10)$d=\psi_{emp}/\sqrt{MS_{within}}$ which corresponded to a medium to large effect size [33]. To draw statistical inferences on between-group differences, in the following step, individual comparisons of group discrimination performance as a function of expertise level was conducted by means of statistical contrasts (Tables F and G in S2 File). A significant difference for the single comparison resulted in only one significant difference between the group of music teachers/musicologists (*μ*_*2*_) and conductors/orchestra musicians (*μ*_3_) (see Table G in S2 File). As a consequence of the contrast analyses, the 5 groups with different levels of self-reported sound discrimination expertise were collapsed into only 2 groups displaying low and high sound-discrimination expertise (Table H in S2 File). The "low" group was comprised of music teachers/musicologists and non-musicians/amateur musicians; the "high" group was comprised of composers/arrangers, producers/audio engineers and conductors/orchestra musicians. A significant difference between groups for the *d'* values was found (*t*(600) = -7.93; *p* < .001; *d* = 0.68; 95% *CI* [0.51, 0.86]). Additionally, the influence of familiarity with the composition (as identified by the correct naming of the title and composer's name) was controlled for. Familiarity had a significant influence on the discrimination performance (see Table I in S2 File and Fig 1c). Finally, the correct responses for the groups of low expertise/no familiarity and high expertise/familiarity (see Table J in S2 File) were calculated and revealed a clear tendency: Participants with a high sound discrimination expertise who were familiar with the composition reached 84.7% of correct responses and outperformed those participants who were unfamiliar with the composition and had low sound discrimination expertise (65.46% correct responses).

## Results and Discussion

In a first step, the sensitivity value of *d* prime (*d'*) [21] and the response bias *c* were calculated (see Fig 1a and 1b for the overall distribution of discrimination performance and response bias). On average, participants discerned above chance (*d'* > 0), with a mean value of *d'* = 1.62, between the sound sources (*t*[601] = 35.8, *p* < .001, Cohen’s *d* = 1.45). Next, we investigated whether participants showed a response behavior in favor of a preference for OSL or LOR. In detail, a negative value of the response bias *c* (*c < 0*) would have indicated an answering bias in favor of the OSL, whereas a positive value would indicate a preference for the LOR. The response bias (Fig 1b) was centered on 0 and did not therefore show any significant tendency in favor of a preference for OSL or LOR.

The length of the stimuli showed a significant correlation with neither the *d*’ value (*r*(10) = -.471, *p* = .17) nor the response bias *c* (*r*(10) = -.01, *p* = .79). However, as shown in Fig 1c, the listeners’ expertise in sound discrimination, as indicated by self-reported occupational activities and collapsed into two groups with low sound-discrimination expertise (non-musicians, amateur musicians, musicologists, and music teachers) and high sound-discrimination expertise (orchestra musicians, audio engineers, conductors, composers, and arrangers) (see Tables D and H in S2 File), had a significant influence on their discrimination performance. As expected, participants with high sound-discrimination expertise achieved a discrimination performance of *d’* = 2.09 and a correct response rate of 80,0% and outperformed those with low expertise (*d’* = 1.37, correct response rate = 68.57%; between-groups difference for *d’*: *t*(600) = -7.93; *p* < .001; *d* = 0.68; 95% CI [0.51, 0.86]). Detailed information on between-groups differences of high vs. low sound discrimination expertise is given in Table H in S2 File.

A positive correlation between the degree of musical sophistication as measured by the Goldsmiths Musical Sophistication Index (*Gold-MSI*, *General Factor*) [31] and discrimination performance *d*’ was found (*r*(602) = 0.27, *p* < .001) as well as between the Gold-MSI and response bias *c* (*r*(602) = -.12, *p* = .001). Thus, the higher the level of musical sophistication, the stronger was the response bias in favor of OSL as the suspected sound source.

The analysis of correct response rates (Fig 1d) revealed that listeners identified the OSL and LOR versions with an overall correct response rate of 72.5%. However, the OSLs passed Turing’s 70% criterion only when the group of listeners with low sound-discrimination expertise had to decide on the source of sounds; their correct responses remained under 70% (for descriptive values, see Table H in S2 File). Discrimination performance was also positively influenced by familiarity with the composition (Table I in S2 File). Put differently, those listeners who had less listening expertise and were less familiar with a particular composition were less likely to correctly identify the sound sources (Table J in S2 File). Additionally, analyses also revealed large differences in discrimination performance among the 10 musical sections: As shown in Table K in S2 File, discrimination performance varied between the lowest value of *d’* = 0.85 for score section #9 and the highest value of *d’* = 1.93 for score section #2. However, reasons for differences in discriminability on the level of score sections must be provided by future studies. Potential explanations could be based on the instrumentation of the particular section that might offer additional cues for the identification of the sound source. Finally, the influence of the different types of headphones used by participants might have influenced the results. The extent of this influence would have to be clarified in future studies, but is an unavoidable intervening variable in this type of internet experiment. However, the technical demands posed by the audio equipment were not extraordinary in this study; additionally, loudness was controlled for by counting the correct the number of timpani beats before the start of the experiment.

## Conclusions

Although the average discrimination performance was clearly above chance, less experienced listeners fell for the successful imitation of human behavior, satisfying Turing's criterion of 70%. In other words, the OSL often generated the impression of a human-controlled sound source for this group. When considering the numerous acoustical cues generated by the highly differentiated use of instruments in the complex score of Stravinsky's *The Rite of Spring*, the average finding of 72.5% of correct responses likely overestimates the true discrimination performance of a "normal" (non-expert) subject listening to musical examples from a less differentiated genre (e.g., a musical or film music). In comparison, current research on applications of artificial intelligence in art production [34] demonstrated that computer-generated paintings came close to the style of famous human painters—at least for non-experts. Findings from this study suggest that in the near future, the increasing expertise of audio engineers in creating OSL arrangements and the application of computer algorithms in the generation of musical expression will help to improve the quality of digital orchestras. Given financial constraints in production, it is likely that high quality sound libraries will become more common in the production of music [35].

## Supporting Information

S1 File **Fig A. Process of participant selection from the initial total number of *N* = 1,563 responses to *N* = 602 valid cases. Fig B. Coding fields for the SDT analysis. Fig C. Workflow of iterative stimulus optimization and evaluation. Fig D. Control for loudness matching between OSL and LOR music examples for the 10 selected score sections**. Loudness analysis was conducted by means of the software *dBSONIC* [36] (y-axis: loudness in sone [soGF], red line: Berlin Philharmonic, green line: Orchestra Sample Library). For description of score sections, see **Table B** in S2 File. **Fig D. Coding fields for the SDT analysis.**
(DOCX)

S2 File**Table A. Illustration of the iterative optimization of passages from Igor Stravinsky’s *The Rite of Spring* by means of the OSL sample library (Sound Example No. 1).** Transcription of comments on different OSL versions of the same score section given by three conductors, when comparing the Orchestra Sample Library (OSL) versions to the Live Orchestra Recording (LOR) version. The iterative procedure was stopped when no more major suggestions for improvements of the OSL version were made. This was the case after the second round of conductors’ comments. In the final (third version) only smaller corrections were made, and OSL and LOR versions were matched for loudness. **Table B. Sample description (valid cases only; values in brackets indicate 1 SE).** *Gold-MSI* = score from the Goldsmith Sophistication Index/General Sophistication Factor [31]. This questionnaire is based on 15 items, answered on a 7-point Likert scale. Higher scores indicate a higher degree of general musical sophistication (max. = 105). However, the ANOVA omnibus test for the degree of general musical sophistication resulted in a significant overall difference between groups (*F*(4,597) = 33.84, *p* < .001, *η*^2^ = 0.18). Conductors/orchestra musicians ranked highest, while non-musicians/amateur musicians showed the lowest sophistication scores. **Table C. Selected passages from Igor Stravinsky's orchestral work *The Rite of Spring* (1913).** Extracts from live orchestra recordings are based on the CD: Rattle, S. (Conductor). (2013). *Stravinsky—Le Sacre du Printemps* [Recorded by the Berlin Philharmonic]. Warner Classics 7236112. **Table D. Signal Detection Analysis of responses for groups of different sound-discrimination expertise (means and SE).** Negative values indicate response bias in favor of the OSL; positive values indicate response bias in favor of the LOR. **Table E. Results of between group analyses (ANOVA) for discrimination performance (*d'*) as a function of expertise level**. Effect size *η*^2^ = 0.10. **Table F. Contrasts and corresponding statistical hypotheses reflecting an increasing average discrimination performance as a function of expertise level.** *μ*_1_ = Non-musicians/amateur musicians, *μ*_*2*_ = music teachers/musicologists, *μ*_3_ = conductors/orchestra musicians, *μ*_4_ = producers, audio engineers, *μ*_5_ = composers/arrangers. **Table G. Statistical tests for the contrasts.** * *p* < .0125 (one-tailed), adjusted *p* value for multiple comparisons (Bonferroni); *t*_*crit*.,*df* = 597, *p* = .0125_ = 2.247; the effect size of each contrast is calculated as the standardized difference of means ($d=\psi_{emp}/\sqrt{MS_{within}}$) (p. 173 in [37]). **Table H. Signal detection analysis of responses for the two resulting groups of low and high sound-discrimination expertise (means and SE). Table I. Discrimination performance (means and standard errors) as a function of familiarity with the test composition.** Only answers with "yes" responses and the correct naming of the piece and composer were considered; all differences were significant at the *p* < .001 level; *t*-test for between-groups differences of *d'* values: *t*(562) = -8.11; *p* < .001; *d* = 0.50; 95% *CI* [0.33, 0.67]. **Table J. Comparison of groups of sound discrimination expertise (high vs. low) and familiarity with the composition (no vs. yes) for percentage of correct responses (means and SE). Table K. Signal detection analysis of responses for each of the 10 selected musical sections.** Negative values indicate response in favor of the OSL; positive values indicate response in favor of the LOR.(DOCX)
