# Correction: Replacing the Orchestra? - The Discernibility of Sample Library and Live Orchestra Sounds

**DOI:** 10.1371/journal.pone.0161911

**Published:** 2016-08-25

**Authors:** Reinhard Kopiez, Anna Wolf, Friedrich Platz, Jan Mons

Fig 1 contains an error. The distributions as well as the legend in panel C are absent from the current version. Please find the corrected version here.

**Fig 1:**
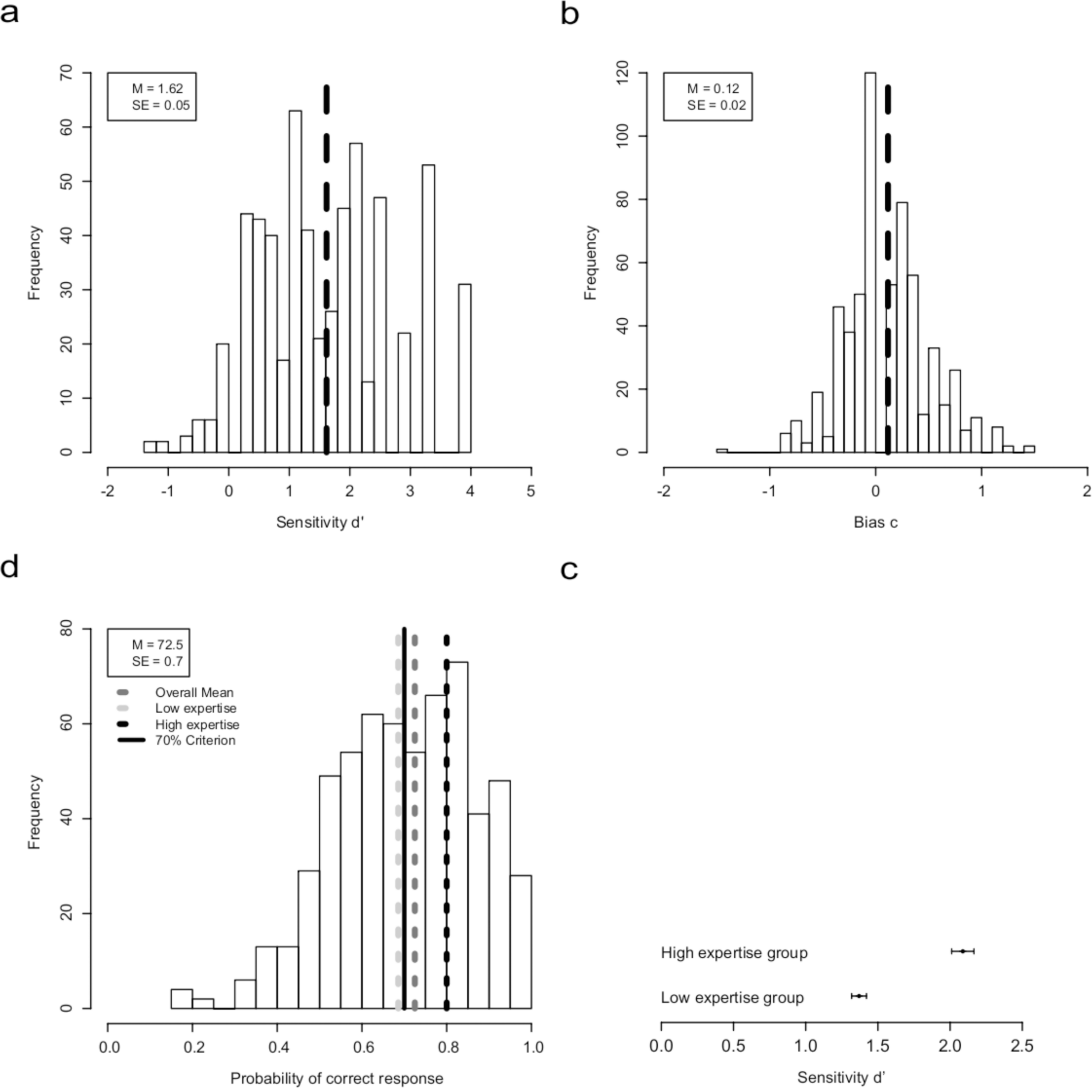
Results from the auditory discrimination task between orchestra sample library and live orchestra recording. (a) Histogram of the overall sensitivity (N = 602) in the discrimination of orchestra sample libraries (OSL) and live orchestra recordings (LOR) in a single-choice paradigm. The dashed line represents the mean discrimination performance, whereas the value of 0 indicates discrimination at chance level. (b) Distribution of response bias with a mean close to 0. Negative values indicate answering response in favor of the OSL; the positive values indicate answering response in favor of the LOR. (c) Discrimination performance for groups of low sound-discrimination expertise (non-musicians, amateur musicians, musicologists, and music teachers) and high sound-discrimination expertise (orchestra musicians, audio engineers, conductors, composers, and arrangers). (d) Correct response rates (hits and correct rejections) for the total sample (72.5%), the subgroups of low vs. high sound discrimination expertise (68.6% vs. 80.0%), and Turing's criterion of 70% for correctly identifying the sound sources to prove AI. Only the group with low sound-discrimination expertise was “cheated” more easily by the samples in that they could not identify correctly the sound source above a rate of 70%.

There is an error in the ‘Data Analysis’ subsection of the Materials and Methods section. The second sentence of the second paragraph contains an incorrect equation. The correct equation should be: (F(4) = 17.35, p < .001, = 0.10)
