# Supplementary material for: Replacing the Orchestra? – The Discernibility of Sample Library and Live Orchestra Sounds
Source: PLoS One. 2016 Jul 6;11(7):e0158324. doi: 10.1371/journal.pone.0158324 (PMC4934781; doi:10.1371/journal.pone.0158324)
Supplement: S1 File — Fig A. Process of participant selection from the initial total number of N = 1,563 responses to N = 602 valid cases. Fig B. Coding fields for the SDT analysis. Fig C. Workflow of iterative stimulus optimization and evaluation. Fig D. Control for loudness matching between OSL and LOR music examples for the 10 selected score sections. Loudness analysis was conducted by means of the software dBSONIC [36] (y-axis: loudness in sone [soGF], red line: Berlin Philharmonic, green line: Orchestra Sample Library). For description of score sections, see Table B in S2 File. Fig D. Coding fields for the SDT analysis. (DOCX) [file pone.0158324.s001.docx]

**Supporting Information**

**S1 File**

**Fig A*.* Process of participant selection from the initial total number of *N* = 1,563 responses to *N* = 602 valid cases.**


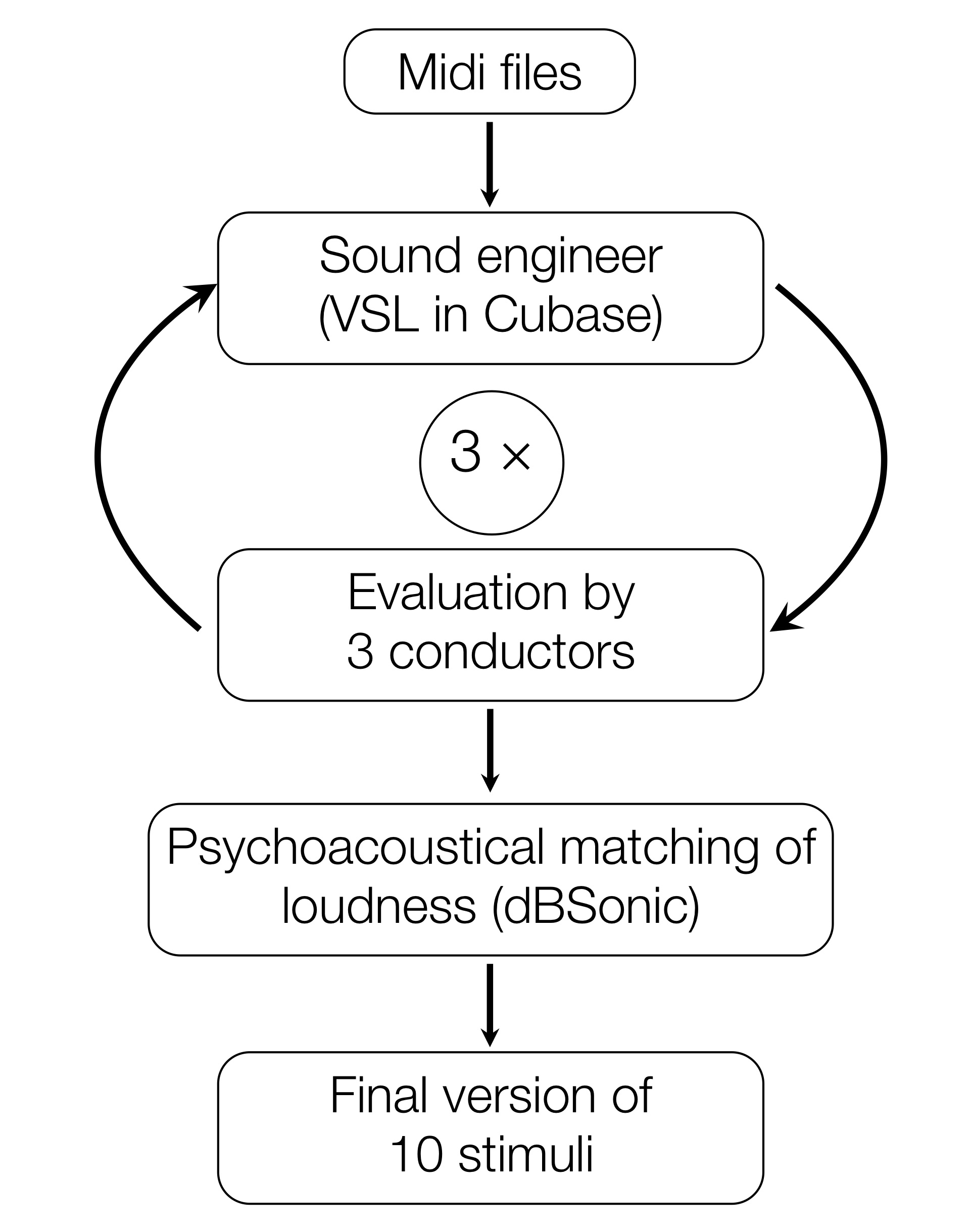


**Fig B. Workflow of iterative stimulus optimization and evaluation.**


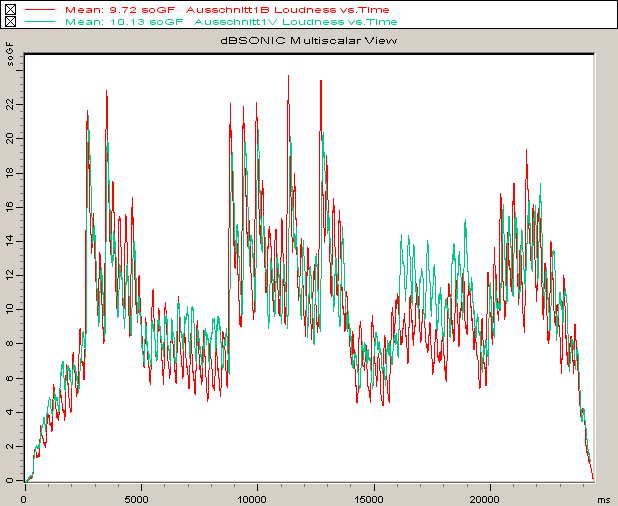


Score section 1.


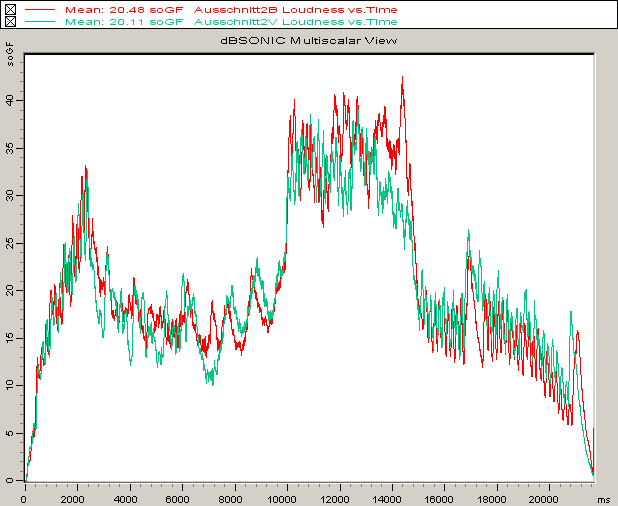


Score section 2.


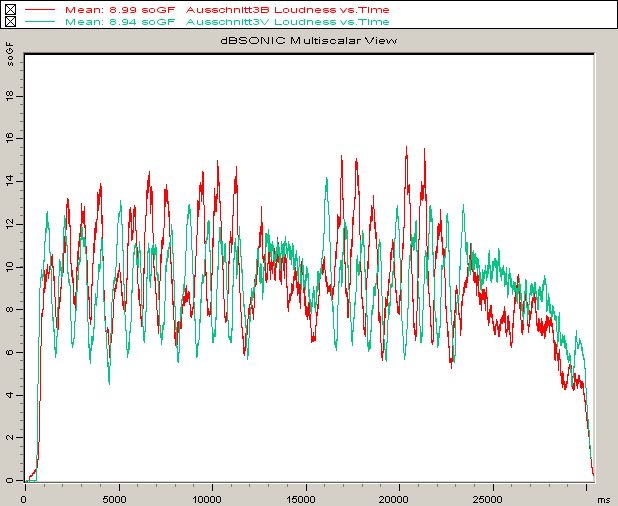


Score section 3.


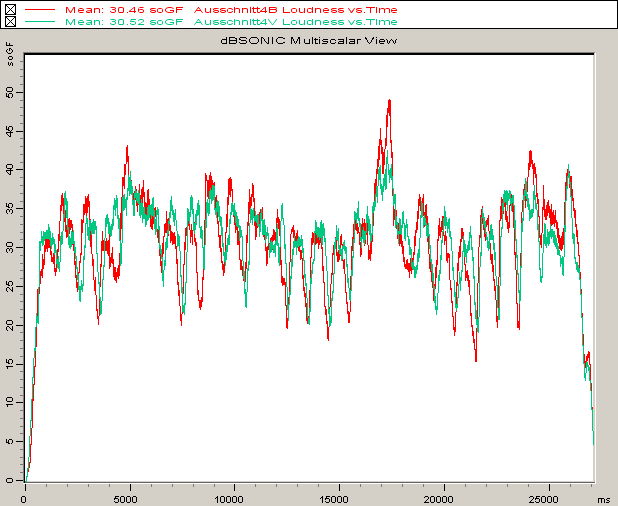


Score section 4.


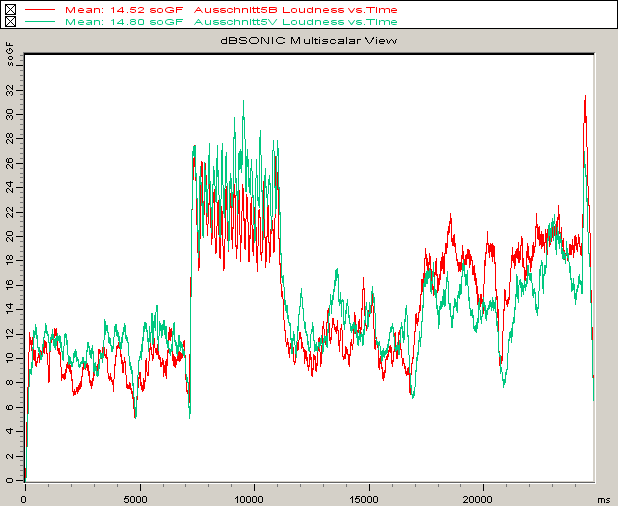


Score section 5.


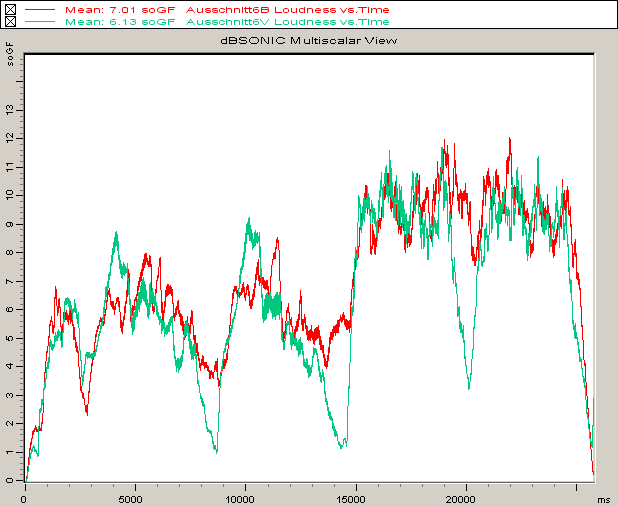


Score section 6.


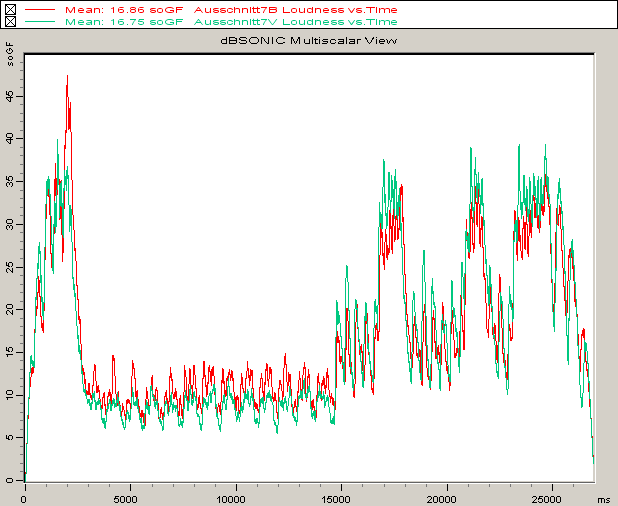


Score section 7.


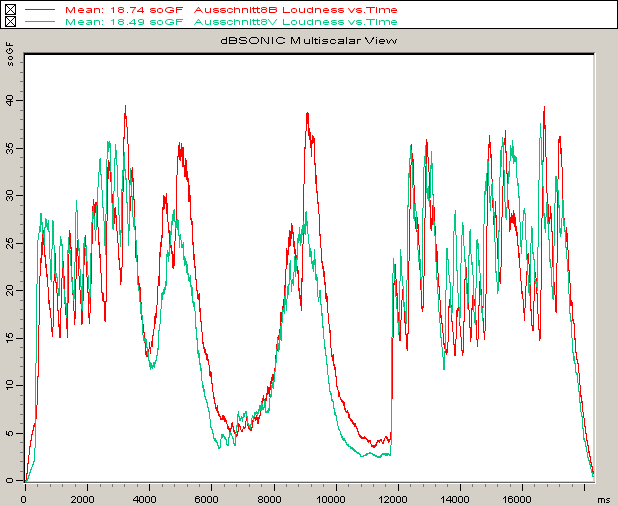


Score section 8.


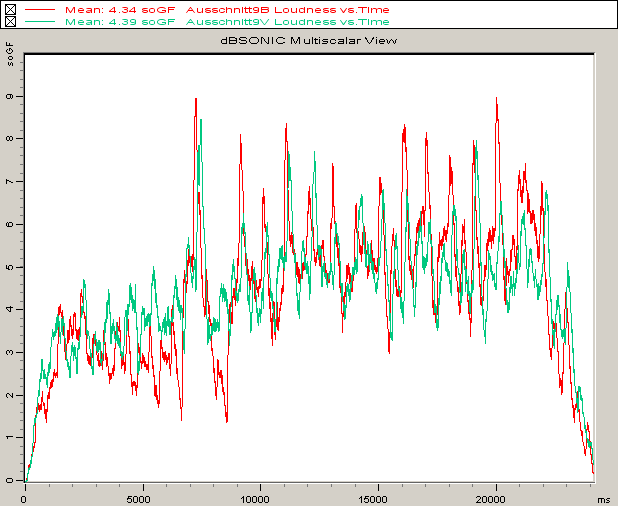


Score section 9.


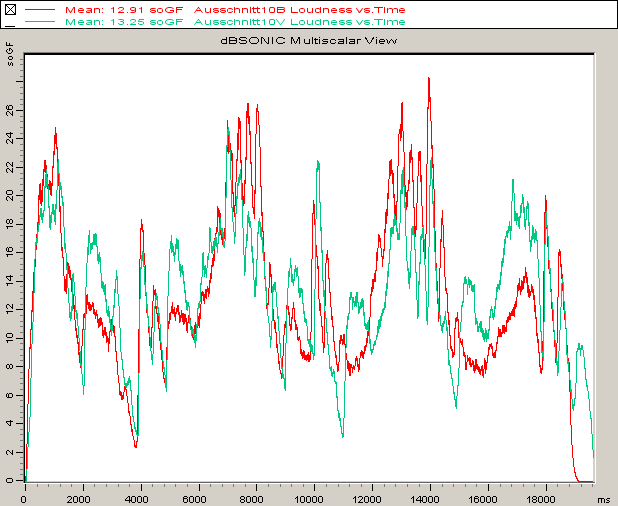


Score section 10.

**Fig C. Control for loudness matching between OSL and LOR music examples for the 10 selected score sections**.

**Fig D. Coding fields for the SDT analysis.**
