# Supplementary material for: Replacing the Orchestra? – The Discernibility of Sample Library and Live Orchestra Sounds
Source: PLoS One. 2016 Jul 6;11(7):e0158324. doi: 10.1371/journal.pone.0158324 (PMC4934781; doi:10.1371/journal.pone.0158324)
Supplement: S2 File — Table A. Illustration of the iterative optimization of passages from Igor Stravinsky’s The Rite of Spring by means of the OSL sample library (Sound Example No. 1). Transcription of comments on different OSL versions of the same score section given by three conductors, when comparing the Orchestra Sample Library (OSL) versions to the Live Orchestra Recording (LOR) version. The iterative procedure was stopped when no more major suggestions for improvements of the OSL version were made. This was the case after the second round of conductors’ comments. In the final (third version) only smaller corrections were made, and OSL and LOR versions were matched for loudness. Table B. Sample description (valid cases only; values in brackets indicate 1 SE). Gold-MSI = score from the Goldsmith Sophistication Index/General Sophistication Factor [31]. This questionnaire is based on 15 items, answered on a 7-point Likert scale. Higher scores indicate a higher degree of general musical sophistication (max. = 105). However, the ANOVA omnibus test for the degree of general musical sophistication resulted in a significant overall difference between groups (F(4,597) = 33.84, p < .001, η2 = 0.18). Conductors/orchestra musicians ranked highest, while non-musicians/amateur musicians showed the lowest sophistication scores. Table C. Selected passages from Igor Stravinsky's orchestral work The Rite of Spring (1913). Extracts from live orchestra recordings are based on the CD: Rattle, S. (Conductor). (2013). Stravinsky—Le Sacre du Printemps [Recorded by the Berlin Philharmonic]. Warner Classics 7236112. Table D. Signal Detection Analysis of responses for groups of different sound-discrimination expertise (means and SE). Negative values indicate response bias in favor of the OSL; positive values indicate response bias in favor of the LOR. Table E. Results of between group analyses (ANOVA) for discrimination performance (d') as a function of expertise level. Effect size η2 = 0.10. Table F. Contra [file pone.0158324.s002.docx]

**Supporting Information**

**S2 File**

**Table A. Illustration of the iterative optimization of passages from Igor Stravinsky’s *The Rite of Spring* by means of the OSL sample library (Sound Example No. 1).**

| *Time* | *Score section* | *Conductor comments on OSL Version 1* | *Conductor comments on OSL Version 2* |
| --- | --- | --- | --- |
| 0 s | 19 |  | Remarkably rhythmical, exciting |
| 04 s | 3 bars after 19 |  | Needs more “noise” from the string instruments |
| 05 s | 4 bars after 19 |  | Notes could be shorter (staccato) |
| 06 s | 5 bars after 19 | Double bassoon too loud |  |
| 08 s | 7 bars after 19 | Add breathing noise to woodwinds, more note sustain to be added | More time between first forte note after piano section |
| 14 s | 4 bars after 20 | Sound of strings is too “thin“ |  |
| 19 s | 21 | Bassoon 1 and 2 should be more prominent | Grace-note should be closer to main note |
| 21 s | 2 bars after 21 | Oboe should be less prominent |  |
| **Summary of comments by conductors** | |  | Strings are very well characterized; higher precision than in a live orchestra; OSL version sounds better than LOR version. |

**Table B. Sample description (valid cases only; values in brackets indicate 1 SE).**

| *Group* | *N* | *Age* | *Sex (m/f)* | *Gold-MSI* |
| --- | --- | --- | --- | --- |
| Composers/  Arrangers | 126 | 32.79  (0.44) | 123/3 | 102.10  (1.24) |
| Producers/Audio engineers | 42 | 32.17  (1.31) | 40/2 | 101.57  (1.81) |
| Conductors/Orchestra musicians | 38 | 36.58  (1.74) | 35/3 | 106.16  (1.66) |
| Music teachers/ Musicologists | 125 | 32.86  (1.04) | 72/52 | 101.60  (0.95) |
| Non-musicians/  Amateur musicians | 271 | 31.25  (0.69) | 193/78 | 87.75  (1.14) |
| Total sample | 602 | 32.31  (0.44) | 463/139 | 95.75  (0.69) |

**Table C. Selected passages from Igor Stravinsky's orchestral work *The Rite of Spring* (1913).**

| *Sec-tion #* | *CD track* | *Time position* | *Length (s)* | *Position in score* | *Musical features* |
| --- | --- | --- | --- | --- | --- |
| 1 | 02 | 0.52-1.14 | 0.24 | II, 2 before 20 | Famous, loud *Bombo* theme played by all strings, various use of brass instruments. |
| 2 | 04 | 0.41-1.02 | 0.21 | IV, 2 before 44 | French horn solo with interesting spatial sound and selective accompaniment by strings, followed by common *staccato* of all woodwind and brass instruments. |
| 3 | 05 | 0.27-0.57 | 0.30 | V, from 49 | Heavy, sluggish theme played by strings (*sostenuto e pesante*), soft with short entries of oboe and bassoon. |
| 4 | 05 | 2.04-2.31 | 0.27 | V, from 53 | Same theme as Section 3, but this time loud and nearly *tutti* with support from percussion section. |
| 5 | 06 | 0.33-0.57 | 0.24 | VI, upbeat to 60 | Initially soft *legato* theme of woodwind, followed by loud *staccato* response by all strings. Afterwards, a *legato* theme of trumpets and flutes, followed by *legato* theme played by high strings. |
| 6 | 11 | 1.28-1.57 | 0.29 | XI, from 99 | Soft *cantabile* theme, initially performed by french horn, followed by strings in *mezzo-forte*. |
| 7 | 12 | 0.48-1.16 | 0.28 | XII, 2 before 114 | Soft *pizzicato* of all strings together with timpani and few wood winds; short intersections by brass instuments. |
| 8 | 13 | 0.03-0.21 | 0.18 | XIII, 3 after 121 | Rhythmical, accentuated fanfare by wind instruments with response from wind, followed by timpani solo. |
| 9 | 14 | 0.57-1.27 | 0.31 | XIV, from 131 | Continuous *legato* of alto flute combined with *staccato* of bassoons, followed by brass solo. |
| 10 | 14 | 1.55-2.14 | 0.19 | XIV, before 135 | Interplay between woodwind and other instrument groups. |

**Table D. Signal Detection Analysis of responses for groups of different sound-discrimination expertise (means and SE).**

| *Group* | *d'* | *c* | *p*_Hit_ | *p*_FA_ | *Correct responses (%)* |
| --- | --- | --- | --- | --- | --- |
| Composers/Arrangers | 2.21  (0.05) | -0.04  (0.04) | 0.82  (0.02) | 0.19  (0.01) | 81.75  (1.29) |
| Producers/Audio engineers | 1.80  (0.16) | 0.15  (0.05) | 0.73  (0.03) | 0.18  (0.02) | 76.31  (2.20) |
| Conductors/Orchestra musicians | 2.00  (0.16) | 0.10  (0.07) | 0.78  (0.03) | 0.19  (.03) | 78.29  (2.32) |
| Music teachers/ Musicologists | 1.45  (0.09) | 0.13  (0.04) | 0.69  (0.02) | 0.23  (0.01) | 69.84  (1.47) |
| Non-musicians/Amateur musicians | 1.33  (0.06) | 0.18  (0.02) | 0.65  (0.01) | 0.24  (0.01) | 67.99  (0.97) |
| Total sample | 1.62  (0.05) | 0.12  (0.02) | 0.70  (0.01) | 0.22  (0.16) | 72.48  (0.67) |

**Table E. Results of between groups analyses (ANOVA) for discrimination performance (*d'*) as a function of expertise level**.

| *Source* | *SS* | *df* | *MS* | *F* | *p* |
| --- | --- | --- | --- | --- | --- |
| Between groups | 76.77 | 4 | 19.19 | 17.35 | < .001 |
| Within groups | 660.39 | 597 | 1.106 |  |  |
| Total | 737.16 | 601 |  |  |  |
|  | | | | | |

**Table F. Contrasts and corresponding statistical hypotheses reflecting an increasing average discrimination performance as a function of expertise level.**

| Contrasts | $H_{1}$ |
| --- | --- |
| $\psi_{1}=\mu_{2}-\mu_{1}$ | $H_{1,a}:\psi_{1}>0$ |
| $\psi_{2}=\mu_{3}-\mu_{2}$ | $H_{1,b}:\psi_{2}>0$ |
| $\psi_{3}=\mu_{4}-\mu_{3}$ | $H_{1,c}:\psi_{3}>0$ |
| $\psi_{4}=\mu_{5}-\mu_{4}$ | $H_{1,d}:\psi_{4}>0$ |
|  | |

**Table G. Statistical tests for the contrasts.**

| Contrasts | $\psi_{\text{emp}}$ | $t$ | $d$ |
| --- | --- | --- | --- |
| $\psi_{1}=\mu_{2}-\mu_{1}$ | 0.126 | 1.108 | 0.12 |
| $\psi_{2}=\mu_{3}-\mu_{2}$ | 0.549 | 2.817**^*^** | 0.52 |
| $\psi_{3}=\mu_{4}-\mu_{3}$ | -0.200 | -0.849 | -0.19 |
| $\psi_{4}=\mu_{5}-\mu_{4}$ | 0.403 | 2.152 | 0.38 |
|  | | | |

**Table H. Signal detection analysis of responses for the two resulting groups of low and high sound-discrimination expertise (means and SE).**

| *Group (sound discrimination expertise)* | *d'* | *c* | *p*_Hit_ | *p*_FA_ | *Correct responses (%)* |
| --- | --- | --- | --- | --- | --- |
| Low | 1.37  (0.05) | 0.16  (0.02) | 0.66  (0.20) | 0.24  (0.01) | 68.57  (0.81) |
| High | 2.09  (0.08) | 0.03  (0.03) | 0.79  (0.01) | 0.19  (0.01) | 80.00  (1.01) |
| Total sample | 1.62  (0.05) | 0.12  (0.02) | 0.70  (0.01) | 0.22  (0.16) | 72.48  (0.67) |

**Table I. Discrimination performance (means and standard errors) as a function of familiarity with the test composition.**

| *Status (familiar with the composition)* | *d'* | *c* | *p*_Hit_ | *p*_FA_ | *Correct responses (%)* |
| --- | --- | --- | --- | --- | --- |
| No (*n* = 354) | 1.34  (0.05) | 0.16  (0.02) | 0.66  (0.01) | 0.24  (0.01) | 68.30  (0.85) |
| Yes (*n* = 210) | 2.09  (0.08) | 0.05  (0.29) | 0.79  (0.01) | 0.18  (0.01) | 79.40  (1.08) |

**Table J. Comparison of groups of sound discrimination expertise (high vs. low) and familiarity with the composition (no vs. yes) for percentage of correct responses (means and SE).**

| *Expertise* | *Familiarity* | *M (%)* | *SE* | *N* |
| --- | --- | --- | --- | --- |
| Low | No  Yes | 65.46  75.04 | 0.94  1.43 | 260  112 |
| High | No  Yes | 76.12  84.27 | 1.56  1.53 | 94  98 |

**Table K. Signal detection analysis of responses for each of the 10 selected musical sections.**

| *Section #* | *d'* | *c* | *p*_Hit_ | *p*_FA_ | *Correct responses (%)* |
| --- | --- | --- | --- | --- | --- |
| 1 | 1.20 | 0.18 | 0.66 | 0.22 | 0.70 |
| 2 | 1.93 | 0.04 | 0.82 | 0.16 | 0.81 |
| 3 | 1.31 | -0.05 | 0.76 | 0.27 | 0.73 |
| 4 | 1.43 | -0.12 | 0.80 | 0.28 | 0.74 |
| 5 | 1.44 | 0.11 | 0.73 | 0.20 | 0.74 |
| 6 | 1.48 | 0.24 | 0.69 | 0.16 | 0.75 |
| 7 | 1.26 | 0.26 | 0.65 | 0.19 | 0.70 |
| 8 | 1.61 | 0.04 | 0.78 | 0.20 | 0.77 |
| 9 | 0.85 | 0.13 | 0.61 | 0.29 | 0.63 |
| 10 | 1.15 | 0.29 | 0.61 | 0.19 | 0.67 |
